# Deep learning based channel estimation method for mine OFDM system

**DOI:** 10.1038/s41598-023-43971-5

**Published:** 2023-10-10

**Authors:** Mingbo Wang, Anyi Wang, Zhaoyang Liu, Jing Chai

**Affiliations:** 1 College of Energy Engineering, Xi’an University of Science and Technology, Xi’an, 710054 China; 2 College of Communication and Information Engineering, Xi’an University of Science and Technology, Xi’an, 710054 China; 3 Shaanxi Energy Institute, Xian Yang, 712000 China

**Keywords:** Electrical and electronic engineering, Computer science

## Abstract

In this paper, we present a channel estimation approach based on deep learning to solve the problem that the orthogonal frequency division multiplexing (OFDM) system channel estimation algorithm cannot accurately obtain the channel state information in the complex environment of the mine, resulting in system performance degradation. First, LS channel estimation matrix is considered as a low-resolution image and the actual channel state information is considered as a high-resolution image. Then the optimization of the LS channel estimation matrix is achieved by the FSRCNN image super-resolution algorithm. We validate the effectiveness of the proposed algorithm by conducting experiments in different channel environments, different number of pilots, and mismatched signal-to-noise ratio scenarios. The simulation results show that the proposed scheme is much better than the traditional LS channel estimation method and the DFT-LS channel estimation method, and the accuracy of the proposed scheme approaches that of the MMSE channel estimation method when the number of pilots is low.

## Introduction

The application of mining 5G system accelerates the process of mine intelligence construction. Orthogonal frequency division multiplexing (OFDM) modulation technology is adopted by the 5G standard for its strong performance against frequency selective fading and high spectrum utilization. However, the interlocking of mine tunnels, the rough and irregular surface of the tunnel walls, the constant switching of operating surfaces, the humid air medium and dust, and many other factors lead to the inability of the receiver to accurately obtain channel state information (CSI), which in turn leads to an increase in the error rate of the wireless communication system and affects the communication quality. As such, it is of practical importance to carry out research on channel estimation of mine OFDM systems.

Traditional channel estimation algorithms include least squares (LS) channel estimation methods and minimum mean square error (MMSE) channel estimation methods^1^. LS channel estimation method is the most commonly used channel method in engineering, which has low computational complexity and does not require prior information of the channel. However, this method cannot eliminate the influence of noise at the pilot. The MMSE channel estimation method has high accuracy, but its computational complexity requires two matrix inversion operations and known channel information, so it is rarely applied in practical systems. Moreover, due to the complexity of the mine environment, channel changes caused by switching of work surfaces and equipment movement will puts a spanner in the practical application of traditional channel estimation algorithms.

In recent years, with the wide application of deep learning in image processing, text recognition, and natural language processing^2–5^, optimizing or replacing traditional channel estimation modules by deep learning algorithms is a current research hotspot. In reference^6^, researchers investigated the application of deep CNN networks in large-scale MIMO-OFDM channel estimation. The model takes advantage of the correlation between adjacent subcarrier channels in OFDM and proposes a channel estimation algorithm called Spatial Frequency CNN (SF-CNN). This algorithm simultaneously inputs the preliminary estimated adjacent subcarrier channel matrices into the CNN for processing. Furthermore, by incorporating the temporal correlation in the time interval channel, the researchers proposed a method called Spatio-Temporal Frequency-Time CNN (SFT-CNN) to further enhance the accuracy of channel estimation. Numerical experimental results demonstrate that the SF-CNN and SFT-CNN methods outperform the non-ideal MMSE estimator in terms of performance and have lower computational complexity. In reference^7^, researchers applied deep learning algorithms to channel estimation in frequency division duplex (FDD) large-scale MIMO-OFDM systems. They employed convolutional neural networks and attention mechanisms to achieve channel estimation while significantly reducing the number of pilot signals. Experimental results demonstrated that this approach outperformed the linear minimum mean square error (LMMSE) channel estimation method. In reference^8^, researchers applied deep learning algorithms to MIMO channel estimation. Experimental results showed that combining compressed sensing networks and differential autoencoder networks for channel estimation outperformed autoencoder-based channel estimation methods in terms of performance. Since the channel estimation process has great similarity with image super-resolution, the literature^9^ considers the time–frequency response of a fast fading communication channel as a two-dimensional image and implements the noise removal using image super-resolution (SR) and image recovery (IR) algorithms, and the simulation results show that the scheme can perform channel estimation efficiently and the results are comparable to those of MMSE channel estimation. Not very much work, however, has been devoted to wireless channel estimation in mines, and major efforts have been directed at Riley channel or the hydroacoustic channel. However, most of the work has focused on the Riley channel or hydroacoustic channel, and not much work has been done on the estimation of wireless channels in mines. Due to the large differences among wireless channels, the relevant estimation models or methods cannot be directly applied to the mine environment, so deep learning-based wireless channel estimation methods for mines need to be investigated.

As such, this paper designs a fast super resolution convolutional neural network (FSRCNN) based OFDM channel estimation method for mines. We implement the optimization of LS channel estimation matrix by FSRCNN to improve the LS channel estimation accuracy. Specifically, the channel response matrix obtained by the LS algorithm is considered as a two-dimensional low-resolution picture, which is then used as the input of the FSRCNN network, and the actual channel state information is considered as a two-dimensional high-resolution picture as the output of the FSRCNN network. Finally, offline training is conducted to optimize the LS channel estimation matrix, making it closer to the real channel state and improving channel estimation accuracy. The contributions of this article are as follows.

- A channel estimation algorithm based on image super-resolution is proposed to address the problem of low accuracy in LS channel estimation algorithm in mine environments. The low-precision channel estimation matrix is treated as a low-resolution image, while the actual channel state information is treated as a high-resolution image. The channel matrix is optimized using image super-resolution algorithms.
- In order to adapt to the unique characteristics of mine environments, this paper proposes a targeted optimization FSRCNN channel estimation algorithm. This algorithm makes specific adjustments to the channel estimation process to improve estimation accuracy. In contrast to previous research^10^, the use of PRelu as the activation function is employed to further enhance the accuracy of channel estimation. By applying the PRelu activation function, it can better adapt to the characteristics of channel estimation tasks and improve estimation accuracy.
- We have conducted extensive simulation experiments to validate the performance of our proposed channel estimation method in the mining environment. These experiments cover various channel conditions, different pilot counts, and scenarios with mismatched signal-to-noise ratios. The experimental results demonstrate that our proposed channel estimation algorithm outperforms the LS channel estimation method significantly. Moreover, under low signal-to-noise ratio conditions, our method performs similarly to the MMSE channel estimation method when using fewer pilots.

The rest of this paper is organized as follows, Section “System model” details the principles of LS and MMSE channel estimation methods. Section “FSRCNN-based channel estimation method” details the design ideas of the FSRCNN-based channel estimation model proposed in this paper. In Section “Simulation and discussion”, simulation experiments under different mines are designed to discuss the performance of the channel estimation method proposed in this paper under different environments. Section “Conclusion” provides conclusions as well as future work.

## System model

Underground coal mines are different from surface channel environments, where many factors such as rough and irregular tunnel wall surfaces, humid air media and dust lead to a very complex wireless channel in the mine tunnel. The commonly used Riley fading cannot accurately characterize the complex wireless environment in mines^11^, so to accurately characterize the communication environment in mines, the Nakagami channel model is chosen, which is able to better simulate the propagation characteristics of electromagnetic waves in complex environments, thus providing more accurate channel simulation results^12^. Its probability density function is shown below.

1 $$f_{R} (a) = \frac{2}{\Gamma (m)}\left( {\frac{m}{\Omega }} \right)^{m} a^{2m - 1} \exp \left( { - \frac{{ma^{2} }}{\Omega }} \right)$$

where $\Gamma (m)$ is the Gamma function, the average power of the multipath scattered field $\Omega = E(a^{2} )$, *m* is known as the fading coefficient in Nakagami channel, it describes the degree of fading of the propagating field caused by scattering process and multipath interference process, it is possible to characterize the different fading properties of the multipath signals by varying the value of *m*^11^.

**Figure 1:**
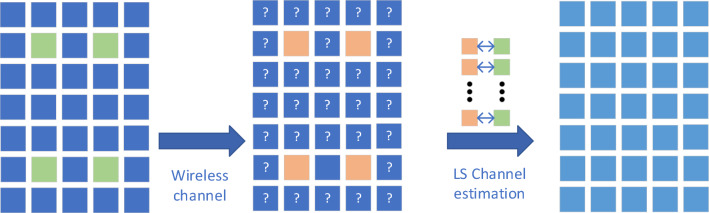
Principle of LS channel estimation.

The signal $X$ sent by the transmitter reaches the receiving end through the wireless channel, and the signal received by the receiving end can be represented by the following formula.

2 $$Y = XH + Z$$

where $$H = [H[0],H[1] \cdots H[N - 1]]^{T}$$ represents the vector matrix of the channel. In the mine environment, each element in matrix *H* is a random variable that follows the Nakagami distribution.$$Z = [Z[0],Z[1] \cdots Z[N - 1]]^{T}[1]$$$$\cdots Z[N - 1]]^{T}$$ represents the vector matrix of the noise, satisfying $$E\{ Z[k]\} = 0$$, $$Var\{ Z[k]\} = \delta^{2}$$, $$k = 0,1, \cdots N - 1$$. For the signal X sent by the transmitter, due to the complexity of the wireless channel, the signal arrives at the receiver end by the interference of the wireless channel. Therefore, it is necessary to estimate the channel state accurately, and combined with the algorithms of channel equalization, demodulation and so on to enable the receiver to accurately recover the information sent by the transmitter. The LS channel estimation method is shown in Fig. 1. It estimates the channel matrix using the pilot information transmitted by the sender. It has low computational complexity and is widely applied.

The basic principle of LS-based channel estimation is to minimize the square of the difference between the received signal and the original transmitted signal. Therefore let $\hat{H}_{LS}$ be the LS estimator of the channel vector H, which corresponds to the cost function shown below.

3 $$\begin{aligned} J(\hat{H}_{LS} ) & = \left\| {Y - X\hat{H}_{LS} } \right\|^{2} \\ & = (Y - X\hat{H}_{LS} )^{H} {(}Y - X\hat{H}_{LS} {)} \\ & = Y^{H} Y - Y^{H} X\hat{H}_{LS} - (\hat{H}_{LS} )^{H} X^{H} Y + \, (\hat{H}_{LS} )^{H} X^{H} X\hat{H}_{LS} \, \\ \end{aligned}$$

In order to minimize the value of the above equation, let its partial derivative with respect to $\hat{H}_{LS}$ be 0.

4 $$\frac{{\partial J(\hat{H}_{LS} )}}{{\partial \hat{H}_{LS} }} = - 2(X^{H} Y)^{H} + 2(X^{H} X\hat{H}_{LS} )^{H} = 0$$

It is possible to obtain $X^{H} Y = X^{H} X\hat{H}_{LS}$, which leads to the solution of the LS estimate.

5 $$\hat{H}_{LS} = (X^{H} X)^{ - 1} X^{H} Y = X^{ - 1} Y = H + X^{ - 1} Z$$

Let $\hat{H}_{LS} (k)$ denote the elements in vector $\hat{H}_{LS}$, where $k = 0,1, \cdots N - 1$. Then the corresponding LS channel estimate for each subcarrier can be expressed as follows.

6 $$\hat{H}_{LS} (k) = \frac{Y(k)}{{X(k)}}$$

As shown in Eq. (6), it can be observed that the LS channel estimation method has a lower computational complexity during the channel estimation process. It estimates the channel by performing a division operation on the received pilot signals only at the positions of the subcarriers carrying the pilot signals. The calculation process does not consider noise and does not require known channel prior information. However, in the complex environment of mining tunnels, various factors such as the interleaving of mining tunnels, rough and irregular surfaces of tunnel walls, humid air medium, and dust make the wireless channel in mining tunnels very complex, with significant multipath effects. LS channel estimation is highly sensitive to noise and multipath effects. When the signal is subjected to noise interference or experiences multipath propagation, LS estimation may introduce significant errors. This leads to inaccurate channel estimation, resulting in reduced system performance.

Hence, in order to improve the issue of low accuracy in LS channel estimation, the MMSE channel estimation method has been proposed. The MMSE channel estimation method is modified for the LS channel estimation method. The correction matrix is assumed to be $w$ and the result in MMSE channel estimation is $\hat{H}_{MMSE} = W\hat{H}_{LS}$. The mean squared deviation of MMSE channel estimation is shown below.

7 $$J(\hat{H}_{MMSE} ) = E\left[ {\left\| {H - \hat{H}_{MMSE} } \right\|^{2} } \right]$$

In MMSE channel estimation, the root-mean-square error can be minimized by choosing a suitable matrix $w$,which can be used to estimate the MMSE channel. Since the error vectors $e = H - \hat{H}_{MMSE}$ and $\hat{H}_{LS}$ are orthogonal, the following derivation can be made.

8 $$\begin{aligned} E[e\hat{H}_{LS}^{H} ] & = E[(H - \hat{H}_{MMSE} )\hat{H}_{LS}^{H} ] \\ & =E[(H - W\hat{H}_{LS} )\hat{H}_{LS}^{H} ] \\ & = E[H(\hat{H}_{LS} )^{H} ] - WE[\hat{H}_{LS} (\hat{H}_{LS} )^{H} ] \\ & = R_{{H(\hat{H}_{LS} )^{H} }} - WR_{{\hat{H}_{LS} (\hat{H}_{LS} )^{H} }} = 0 \\ \end{aligned}$$

Therefore, the correction matrix $w = R_{{H(\hat{H}_{LS} )^{H} }} R_{{\hat{H}_{LS} (\hat{H}_{LS} )^{H} }}^{ - 1}$. where $R_{{H(\hat{H}_{LS} )^{H} }}$ is the mutual correlation matrix, characterizing the similarity between the real channel vector and the estimated obtained channel vector; and $R_{{\hat{H}_{LS} (\hat{H}_{LS} )^{H} }}$ represents the autocorrelation matrix of the LS estimates, which can be derived as follows by combining with Eq. (4).

9 $$\begin{aligned} R_{{\hat{H}_{LS} (\hat{H}_{LS} )^{H} }} & = E[(\hat{H}_{LS} )(\hat{H}_{LS} )^{H} ] \\ & = E[X^{ - 1} Y(X^{ - 1} Y)^{H} ] \\ & = E[(H + X^{ - 1} Z)(H + X^{ - 1} Z)^{H} ] \\ & = E[HH^{H} + X^{ - 1} ZH^{H} + HZ(X^{ - 1} )^{H} + X^{ - 1} ZZ^{H} (X^{ - 1} )^{H} ] \\ & = E[HH^{H} ] + E[X^{ - 1} ZZ^{H} (X^{ - 1} )^{H} ] \\ & = R_{HH} + \delta^{2} (XX^{H} )^{ - 1} \\ \end{aligned}$$

Thus the MMSE channel estimate can be expressed by the following equation.

10 $$\begin{gathered} \hat{H}_{MMSE} = W\hat{H}_{LS} \hfill \\ \, = R_{{H(\hat{H}_{LS} )^{H} }} (R_{HH} + \delta^{2} (XX^{H} )^{ - 1} )^{ - 1} \hat{H}_{LS} \hfill \\ \end{gathered}$$

From Eq. (10), it can be seen that the MMSE channel estimation method is a modification of the LS channel estimation method, which achieves higher computational accuracy. However, the MMSE channel estimation method requires two nested matrix inverse calculations, which will lead to an increase in the computational power of the algorithm and is difficult to implement by hardware devices. Therefore, combined with the above analysis, there is a need to develop a channel estimation algorithm suitable for the complex environment of mines.

## FSRCNN-based channel estimation method

### Algorithm principle

**Figure 2:**
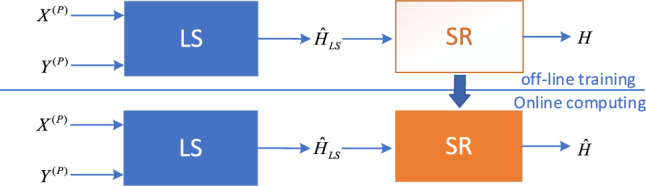
Principle of channel estimation method based on image super-resolution (SR).

In complex mining wireless channel environments, LS channel estimation fails to accurately acquire CSI resulting in a decline in receiver performance. Although the MMSE channel estimation method can improve estimation accuracy, its computational complexity makes it challenging to implement in computer hardware. Given the powerful non-linear mapping capabilities of deep learning algorithms, there has been increasing interest in their application for optimizing channel estimation. Among these approaches, the principle of optimizing LS channel estimation through image super-resolution^13^ is illustrated in Fig. 2.

The optimization of LS channel estimation through image super-resolution algorithm can be divided into two stages. In the offline training stage, the initial low-precision channel estimation result $\hat{H}_{LS}$ is obtained by combining the received pilot signal $Y^{P}$ with the local pilot signal $X^{P}$ using Eq. (6). This result is then used as the input for the image super-resolution network. During the offline stage, the channel response matrix *H* can be generated based on the channel characteristics and model. This generated channel response matrix is then used as the output of the image super-resolution network. In the offline training stage, the image super-resolution network optimizes the set of network parameters using an optimizer. A loss function is employed to evaluate the training results of the network and obtain the optimal set of parameters. In this paper, the mean squared error (MSE) is used as the loss function, which is represented as follows. In the offline training stage, the low-precision channel estimation result and the channel response matrix are treated as the low-resolution and high-resolution images respectively. The improvement of LS channel estimation is achieved through the nonlinear fitting of the neural network.

11 $$L{\text{o}}ss = \frac{1}{M}\sum\limits_{i = 1}^{M} {\mathop {\left\| {[H{\prime} - f([\hat{H}{\prime}_{LS} ],\Theta )} \right\|}\nolimits_{2}^{2} }$$

The equation above represents that *M* denotes the number of sample data included in the training set. $f( \cdot )$ represents the image super-resolution network mapping utilized, and $\Theta$ represents the set of neural network parameters. In addition, for the data input to the neural network, since the LS channel estimation matrix consists of complex numbers, the neural network cannot directly process it. Therefore, we extract the real and imaginary parts of the channel estimation matrix $\hat{H}_{LS}$ and combine them to form a new matrix $\hat{H}{\prime}_{LS}$, which is then input into the neural network. Similarly, in the offline training process, the actual channel response matrix is transformed into matrix $H^{\prime}$.

In online computations, the optimized LS channel estimation method is achieved through the trained network, resulting in improved accuracy of channel estimation. The values obtained through online computations by this network are shown below.

12 $$\hat{H}{\prime} = f(\hat{H}_{LS}{\prime} ,\Theta )$$

By combining the real and imaginary parts of $\hat{H}{\prime}$, the optimized channel matrix $\hat{H}$ is obtained. From this, it can be concluded that selecting an appropriate image super-resolution algorithm is crucial for optimizing LS channel estimation, as it will determine the accuracy of channel estimation.

### FSRCNN channel estimation optimization network

**Figure 3:**
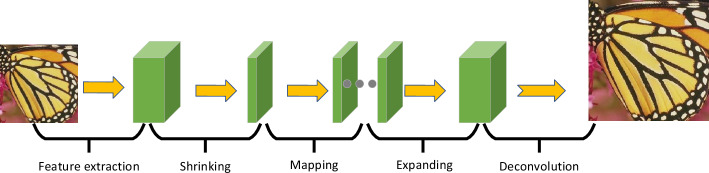
FSRCNN super-resolution model.

The FSRCNN model is an image super-resolution reconstruction model designed by Tang et al.^14^ based on convolutional neural networks. The basic structure of FSRCNN is shown in Fig. 3. The main processes of the FSRCNN model include feature extraction, shrinkage, mapping, expansion, and deconvolution. The functionalities of each process are as follows:

1. Feature extraction module: This module extracts features from the original low-resolution image.
2. Shrinkage layer: Reduces the number of parameters during the transmission process, reducing computational complexity.
3. Mapping layer: Performs nonlinear learning on the image information.
4. Expansion layer: The inverse operation of the shrinkage layer.
5. Deconvolution layer: Uses the previously generated feature maps to upsample and aggregate to obtain the final high-resolution image.

**Figure 4:**
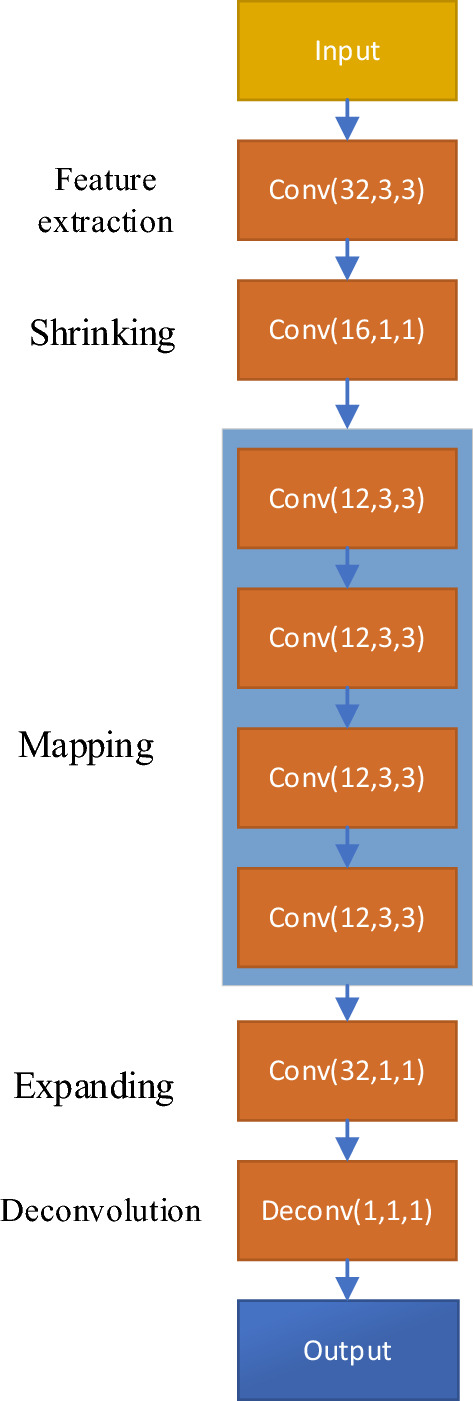
FSRCNN channel estimation neural network structure.

The FSRCNN network structure used in this paper is illustrated in Fig. 4. For the input LS channel estimation matrix, the feature extraction step is performed first. The feature extraction module consists of 32 convolution kernels with a size of 3 × 3. Next, the extracted features are input to 16 convolution kernels with a size of 1 × 1 to achieve feature reduction. After that, the input is passed to the mapping layer, which consists of four convolutional layers with the same structure. Each convolutional layer contains 12 convolution kernels with a size of 3 × 3. Following the mapping layer, the input goes through the expansion layer, which comprises 32 convolution kernels with a size of 1 × 1. It’s worth noting that in the original FSRCNN network, the expansion layer has the same kernel size as the shrinkage layer since it is the inverse operation of the shrinkage layer. However, in this paper, optimization is focused on LS channel estimation matrices, which have a different data type than image data. Therefore, the expansion layer uses convolution kernels with a size of 1 × 1. Finally, the output is obtained through the deconvolution layer.

In addition, the PRelu activation function is used in this paper to avoid the issue of “dead neurons” caused by the Relu function. The Relu and PRelu activation functions are defined as follows. It can be observed from the equation that the PRelu activation function allows negative values in its output. Since the optimized channel matrix obtained from the output of the FSRCNN channel optimization network may also contain negative values, using the PRelu function can better fit the channel estimation matrix and improve the prediction accuracy of the network.

13 $${\text{Re}} lu(x) = \left\{ \begin{gathered} x,x > 0 \hfill \\ 0,x \le 0 \hfill \\ \end{gathered} \right. ,\;\;\;\;\;\;P{\text{Re}} lu(x) = \left\{ \begin{gathered} x,x > 0 \hfill \\ ax,x \le 0,0 < a < 1 \hfill \\ \end{gathered} \right.$$

### Analysis of algorithm time complexity

The FSRCNN channel estimation optimization network model consists mainly of feature extraction, shrinkage, mapping, expansion, and deconvolution modules. The time complexity of each module is as follows:

1. Feature extraction module: For each position in the input channel estimation matrix, 32 convolutions with a size of 3 × 3 are performed, resulting in a time complexity of $O(32 \times h \times w \times 3 \times 3)$, where h and w represent the height and width of the input channel estimation matrix.
2. Shrinkage layer: For each position in the feature map, 16 convolutions with a size of 1 × 1 are performed, resulting in a time complexity of $O(16 \times h \times w \times 1 \times 1)$.
3. Mapping layer: Consisting of 4 convolutional layers, each performing 12 convolutions with a size of 3 × 3 for each position in the feature map. Hence, the time complexity is $O(4 \times 12 \times h \times w \times 3 \times 3)$.
4. Expansion layer: Composed of 32 1 × 1 convolutional kernels, the time complexity is $O(32 \times h \times w \times 1 \times 1)$.
5. Deconvolution layer: The time complexity depends on the size of the output channel estimation matrix.

In summary, the overall time complexity of the FSRCNN network can be approximated as $O(h \times w)$. Since the input to the FSRCNN is a matrix containing the real and imaginary parts, the processed matrix has a length of $N \times 2$, where $N \times 2 = h \times w$. Therefore, the time complexity of the FSRCNN network can be approximated as $O(N)$, where *N* is the length of the matrix. In contrast, the time complexity of the MMSE channel estimation method can be approximated as $O(N^{3} )$. Therefore, it can be concluded that the time complexity of the FSRCNN channel estimation optimization algorithm is much lower than that of the MMSE channel estimation method.

## Simulation and discussion

This chapter discusses the OFDM channel estimation method based on FSRCNN for mining environments. Firstly, the FSRCNN channel estimation model shown in Fig. 4 is implemented using the Keras framework. Then, OFDM symbol waveforms, LS channel estimation matrix, and channel response matrix *H* are generated in the mining environment using Matlab software, and 10,000 training and testing data are generated at different signal-to-noise ratios. The relevant parameters of the OFDM system are shown in Table 1. During the offline training phase, the LS channel estimation matrix is taken as the input and the channel response matrix *H* is taken as the output. The network uses the PRelu function as the activation function, RMSprop as the optimizer, batch = 500, batch_size = 128.

**Table 1:** OFDM system simulation parameters.

Number of subcarriers	64
Number of pilots	16
FFT length	64
Pilot symbol modulation method	QPSK
Number of multipath	5
Wireless channel	Nakagami + AWGN

We selected Nakagami channel to simulate the mine channel environment, set Nakagami channel coefficient m = 0.85 as the mine channel according to previous studies^15,16^, and discussed the performance of FSRCNN based channel estimation method in this channel with different channel environments, different numbers of pilots, and different training signal-to-noise ratios. We have also compared the LS channel estimation method, the MMSE channel estimation method, and the DFT-LS channel estimation method that utilizes the discrete Fourier transform (DFT) as described in reference^17^. Additionally, we have included a comparison of the channel estimation method that utilizes the SRCNN image super-resolution network as described in reference^18^. These comparative analyses have been incorporated into our algorithm comparison experiment to ensure a comprehensive evaluation of various channel estimation techniques. Normalized mean square error (MSE) and bit error rate (BER) are also used as the evaluation criteria for the performance of channel estimation algorithms. Furthermore, for ease of description, we will denote the channel estimation methods based on FSRCNN and SRCNN as FS-CE and SR-CE respectively.

Firstly, we discuss the performance of channel estimation method based on FSRCNN in different channel environments. In this process, we compare the channel estimation performance of different Nakagami channel coefficients *m* and different channel fading parameters under the same coefficient *m*.

**Figure 5:**
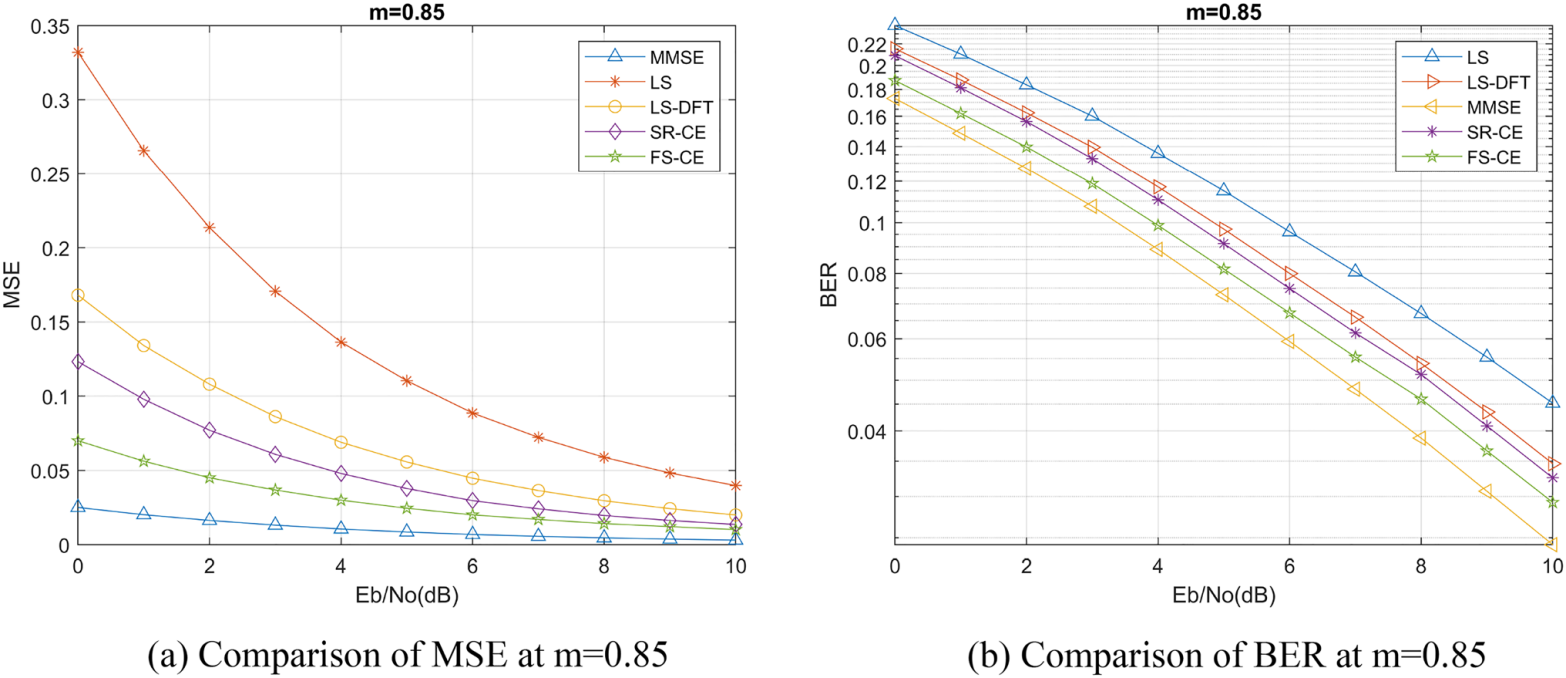
Nakagami coefficient m = 0.85 simulation results.

**Figure 6:**
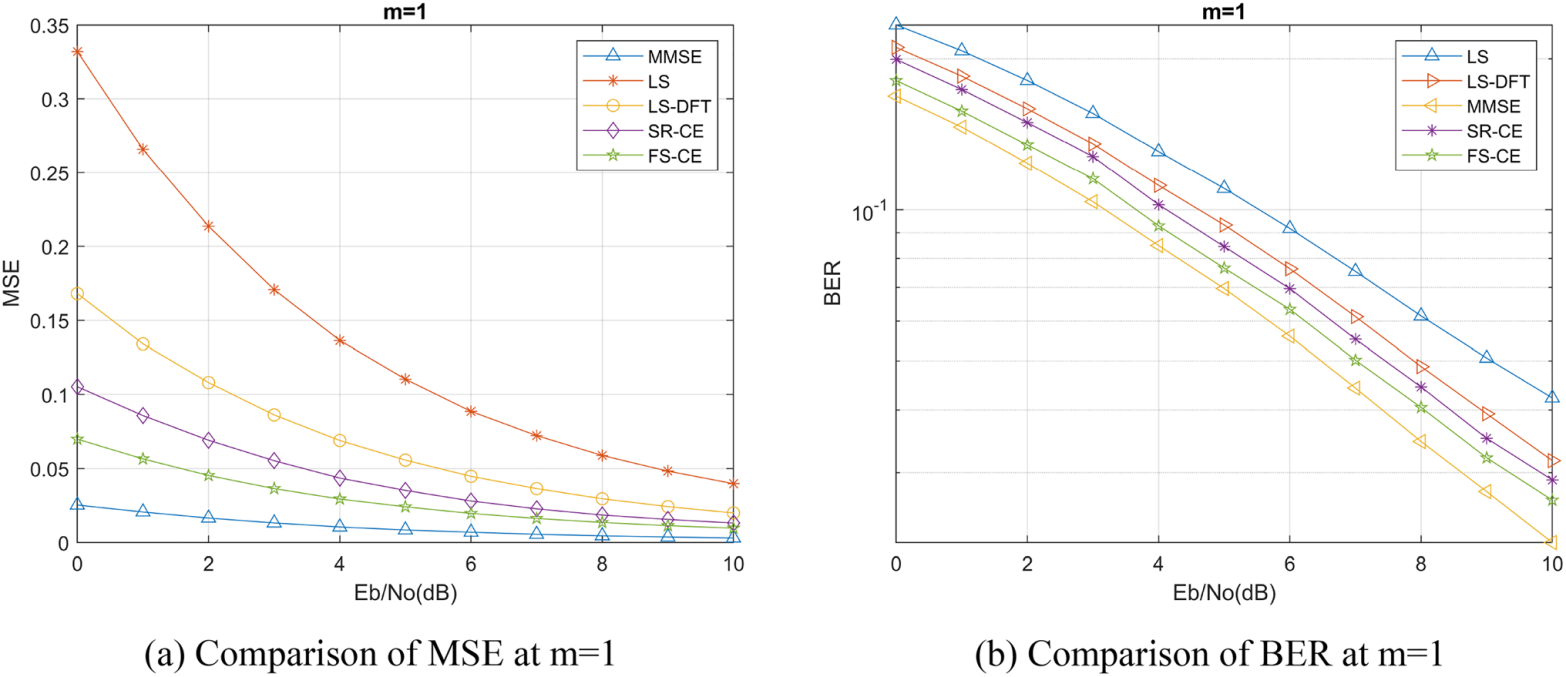
Nakagami coefficient m = 1 simulation results.

**Figure 7:**
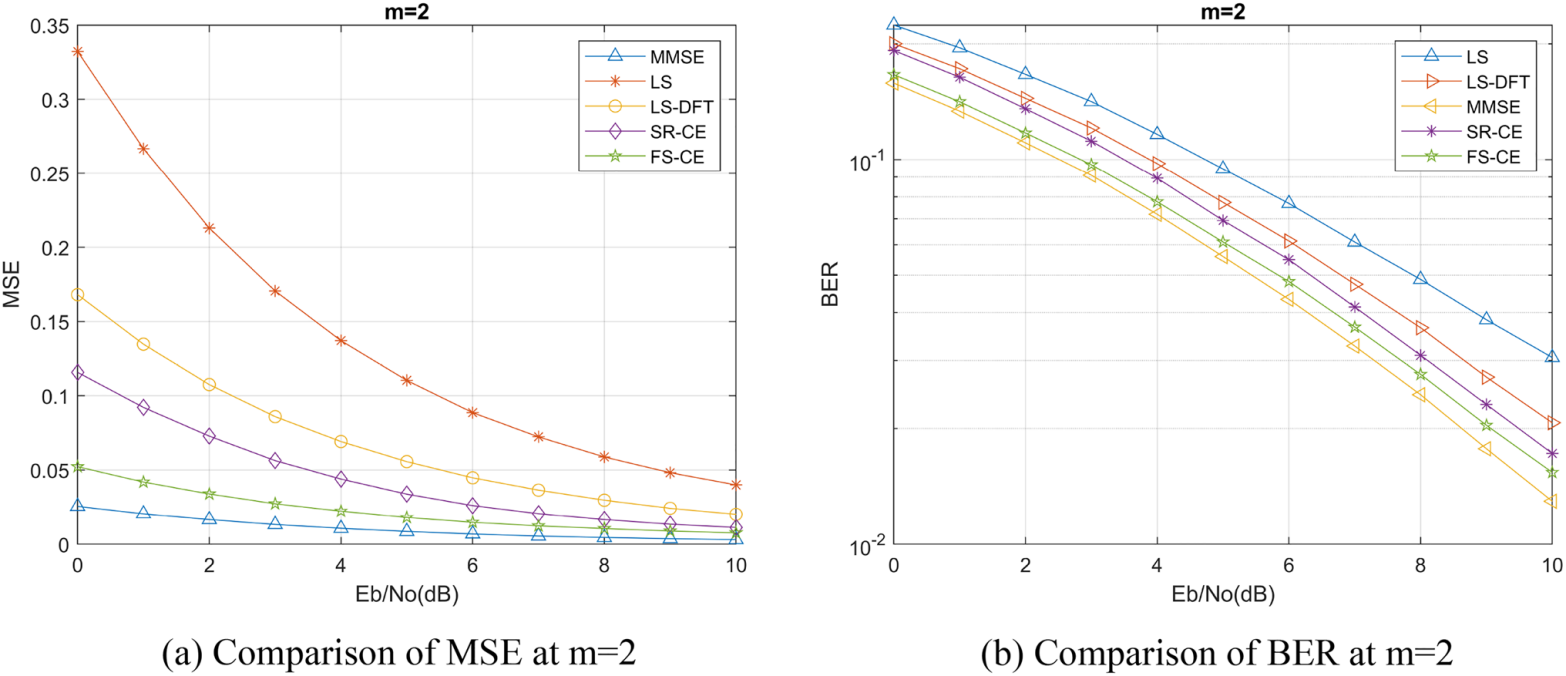
Nakagami coefficient m = 2 simulation results.

The channel estimation accuracy and BER under different Nakagami channel coefficients *m* are shown in Figs. 5, 6, and 7. We can find that the SRCNN and FSRCNN-based channel estimation methods are much better than the traditional LS channel estimation method and the DFT-LS channel estimation method, which indicates that the FS-CE channel estimation method proposed in this paper can achieve the mine channel estimation, and the BER of the communication system using FS-CE channel estimation is lower than that of the traditional LS channel estimation method and the DFT- LS channel estimation methods. For the mining channel with m = 0.85 as shown in Fig. 5b, when the BER = 0.14, the FS-CE algorithm achieves a signal-to-noise ratio gain of approximately 2 dB compared to the LS algorithm, and a signal-to-noise ratio gain of approximately 1 dB compared to the LS-DFT algorithm. Additionally, comparing Figs. 5b, 6b, and 7b, it can be observed that as the Nakagami channel parameter m gradually increases, the system bit error rate decreases, and the difference in bit error rate between the FS-CE channel estimation method and the SR-CE channel estimation method also decreases.

**Figure 8:**
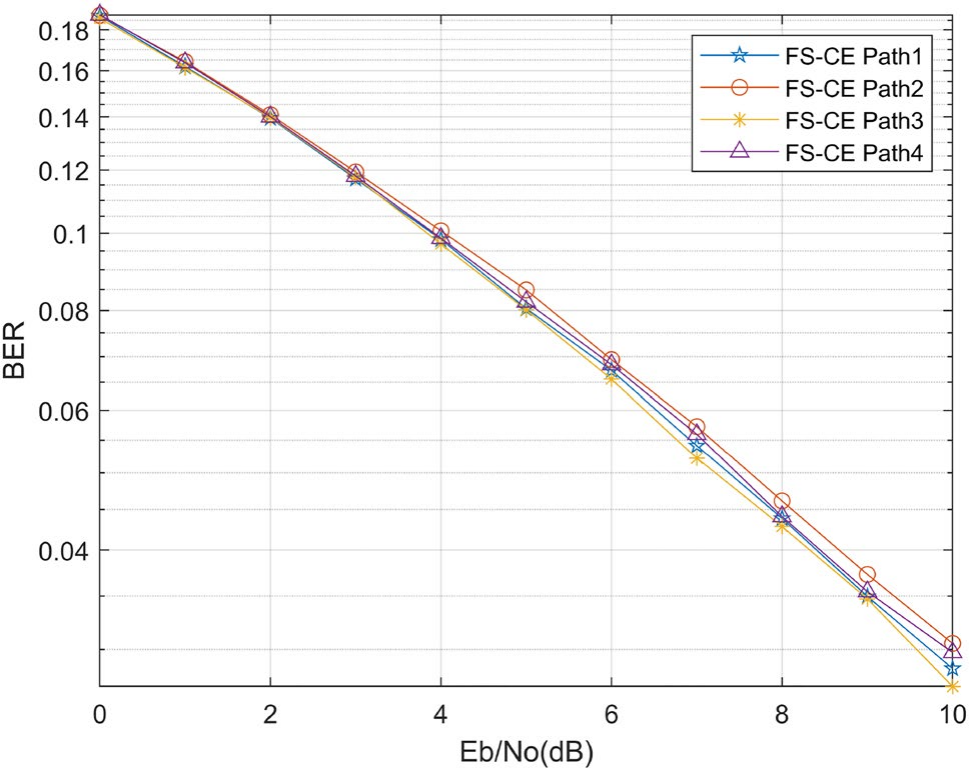
BER curves with different fading parameters.

Then, we analyze the channel estimation algorithm performance under different channel fading parameters. Due to the special environment of the mine channel, multipath fading phenomena such as refraction, reflection, scattering and diffraction are generated during the transmission of electromagnetic waves. Therefore, it is necessary to study the performance of channel estimation algorithms under fading channels. We simulate four different fading coefficients to explore the performance of the FS-CE algorithm. According to the research documented in reference^19^, a study was conducted to design different channel parameters. The design results are presented in Table 2. Figure 8 below shows that the FS-CE channel estimation algorithm proposed in this paper is not affected by multipath fading, and the receiver with FS-CE channel estimation algorithm can work stably and decode accurately in the mine environment.

**Table 2:** Different channel fading parameters.

Channel class	Channel time delay	Channel tap power
Path1	[0 3 5 7 8 11 15]	[0 − 8 − 17 − 19 − 21 − 25 − 30]
Path2	[0 3 4 6 8 11 15]	[0 − 8 − 15 − 17 − 21 − 25 − 30]
Path3	[0 3 9 10 15]	[0 − 8 − 15 − 17 − 21 − 25 − 30]
Path4	[0 1 9]	[0 − 7 − 14]

**Figure 9:**
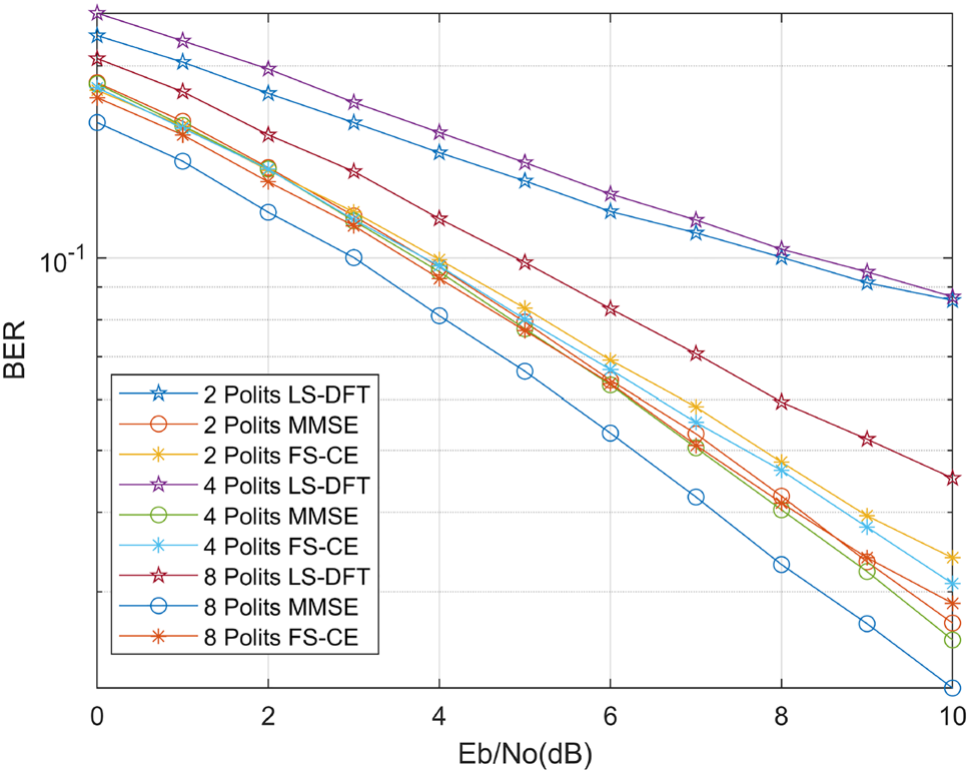
BER curves for different number of polits.

Next, we conducted a comparison of the FSRCNN channel estimation algorithms’ performance across different pilot numbers. Figure 9 illustrates the results. It is apparent that both the DFT-LS and MMSE channel estimation algorithms experience a decline in performance as the number of pilots decreases. However, our proposed FS-CE method exhibits a consistent BER at low signal-to-noise ratios, indicating its resilience to variations in pilot count under such conditions. Furthermore, within the 0–2 dB range, when the pilot count is reduced to 2, the performance of the FS-CE channel estimation method closely matches that of the MMSE algorithm. This suggests that, under low signal-to-noise ratios, the FS-CE method can achieve performance similar to that of the MMSE method by utilizing a reduced number of pilots. Consequently, by leveraging image super-resolution algorithms to optimize the channel estimation method, we not only ensure stable information transmission but also enhance spectral efficiency.

**Figure 10:**
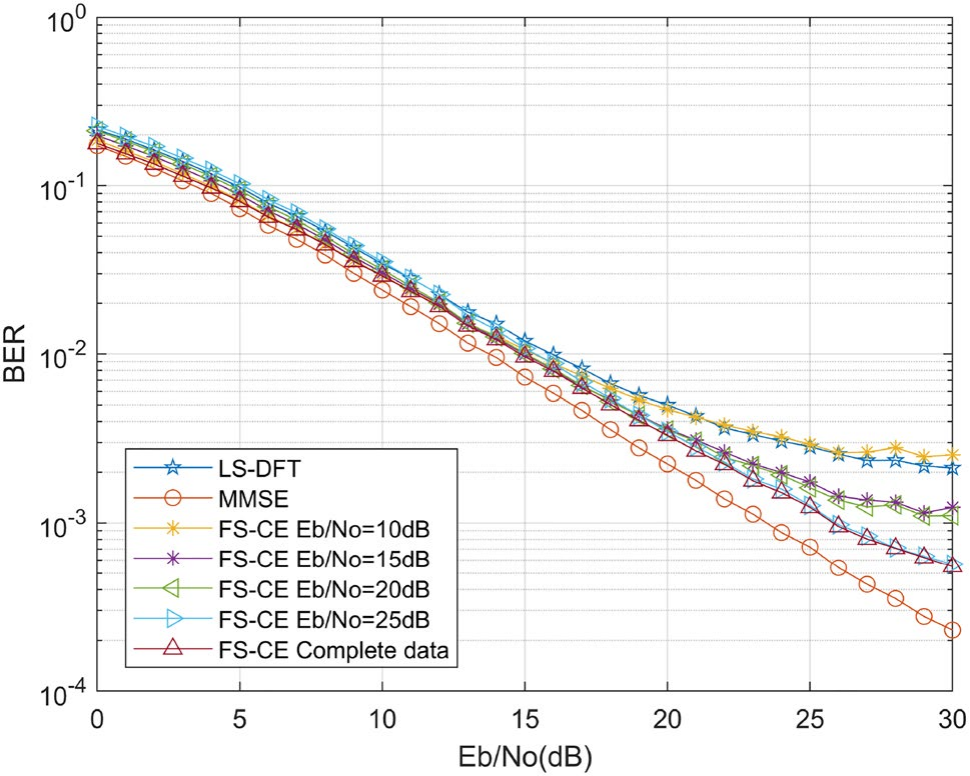
Comparison of algorithm performance under signal-to-noise ratio mismatch.

All offline training phases above were trained using a fixed signal-to-noise ratio, however, in the actual mine environment, the signal-to-noise ratio is variable, thus creating the problem of signal-to-noise ratio mismatch. As such, we generated training data at different signal-to-noise ratios and then trained on the same test set, and the simulation results are shown in Fig. 10 below. As can be seen from the figure below, the accuracy of channel estimation gradually improves as the signal-to-noise ratio of the training data set gradually increases, and the FS-CE channel estimation results approximate the MMSE channel estimation method at 0–5 dB. In addition, we conducted separate training for data at each signal-to-noise ratio, referred to as “Complete data” as shown in the figure below. The experimental results demonstrate that the results obtained from training on data at each individual signal-to-noise ratio are close to those obtained from training at 25 dB. Therefore, in practical mining channel estimation applications, it is possible to select appropriate data at specific signal-to-noise ratios for individual training, without the need to train at all signal-to-noise ratios. This approach saves training time while ensuring channel estimation accuracy.

## Conclusion

To improve the channel estimation accuracy of OFDM system in mining environment, this paper implements the optimization of LS channel estimation by FSRCNN algorithm. In the offline training process, the LS channel estimation matrix is used as the neural network input and the actual channel response matrix is used as the output, and the improvement of LS channel estimation accuracy is achieved by the FSRCNN algorithm. The simulation results show that the FSRCNN channel estimation algorithm based on FSRCNN is much better than the traditional LS channel estimation method and the DFT-LS channel estimation method in the mine wireless environment. Furthermore, when the number of pilot tones is small, the FSRCNN channel estimation algorithm approaches the accuracy of the MMSE algorithm.

The next step of our work will involve collecting datasets from actual mine environments and training different networks based on the real channel conditions. This will allow us to further enhance the accuracy of FSRCNN channel estimation.

## Data Availability

The datasets generated during and/or analysed during the current study are not publicly available due to confidentiality reasons, but are available from the corresponding author on reasonable request.
